# Pentavalent U Reactivity Impacts U Isotopic Fractionation during Reduction by Magnetite

**DOI:** 10.1021/acs.est.3c10324

**Published:** 2024-04-04

**Authors:** Zezhen Pan, Luca Loreggian, Yvonne Roebbert, Barbora Bartova, Myrtille O. J. Y. Hunault, Stefan Weyer, Rizlan Bernier-Latmani

**Affiliations:** † Department of Environmental Science and Engineering, Fudan University, Shanghai 200438, China; ‡ EML, École Polytechnique Fédérale de Lausanne, 1015 Lausanne, Switzerland; § Institute of Eco-Chongming (IEC), Shanghai 200062, China; ∥ Institut für Mineralogie, Leibniz Universität Hannover, D-30167 Hannover, Germany; ⊥ Synchrotron SOLEIL, L’Orme des Merisiers, Saint Aubin BP 48, 91192 Gif-sur-Yvette, France

**Keywords:** pentavalent uranium, isotope fractionation, uranium remediation, redox tracer

## Abstract

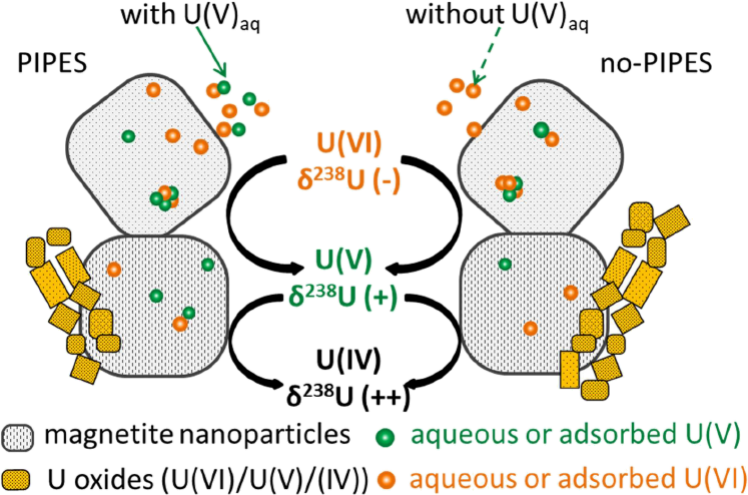

Meaningful interpretation of U isotope measurements relies on unraveling the impact of reduction mechanisms on the isotopic fractionation. Here, the isotope fractionation of hexavalent U [U(VI)] was investigated during its reductive mineralization by magnetite to intermediate pentavalent U [U(V)] and ultimately tetravalent U [U(IV)]. As the reaction proceeded, the remaining aqueous phase U [containing U(VI) and U(V)] systematically carried light isotopes, whereas in the bicarbonate-extracted solution [containing U(VI) and U(V)], the δ^238^U values varied, especially when *C*/*C*_0_ approached 0. This variation was interpreted as reflecting the variable relative contribution of unreduced U(VI) (δ^238^U < 0‰) and bicarbonate-extractable U(V) (δ^238^U > 0‰). The solid remaining after bicarbonate extraction included unextractable U(V) and U(IV), for which the δ^238^U values consistently followed the same trend that started at 0.3–0.5‰ and decreased to ∼0‰. The impact of PIPES buffer on isotopic fractionation was attributed to the variable abundance of U(V) in the aqueous phase. A few extremely heavy bicarbonate-extracted δ^238^U values were due to mass-dependent fractionation resulting from several hypothesized mechanisms. The results suggest the preferential accumulation of the heavy isotope in the reduced species and the significant influence of U(V) on the overall isotopic fractionation, providing insight into the U isotope fractionation behavior during its abiotic reduction process.

## Abstract 2

This work elucidates the role of pentavalent U in abiotic isotope fractionation and is relevant for the application of U isotope fractionation as a redox proxy.

## Introduction

Uranium (U) is a ubiquitous element in the Earth’s crust and a contaminant of concern in subsurface environments. Soluble hexavalent U [U(VI)] can be immobilized via biotic or abiotic reduction to sparingly soluble tetravalent U [U(IV)] in anoxic environments. Ferrous iron-bearing minerals, such as magnetite (Fe_3_O_4_), are abundant in natural sediments and also a product of steel corrosion.^1^ Therefore, they are relevant for contaminated aquifers and nuclear waste disposal. As a result, the reduction of U(VI) by Fe(II)-containing mineral phases has been extensively studied to pinpoint the underlying reduction mechanism(s), such as the roles of the Fe(II)/Fe(III) ratio at the mineral surface,^2,3^ of U(VI) loading,^4^ and of pH and aqueous chemistry.^5,6^ While crystalline uraninite U(IV) has been considered the major abiotic reduction product,^3,4,6,7^ there are several studies documenting the formation and persistence of pentavalent U [U(V)] during U(VI) reduction by forming or dissolving/reprecipitating iron-bearing minerals.^2,6,8−12^ In particular, the incorporation of U(V) in iron oxide mineral phases has been reported during the coprecipitation of U(VI) with magnetite or green rust,^9,10^ the reduction of U(VI) concomitantly with the dissolution and recrystallization of iron oxides,^2,6,8,11,12^ or even within the structure of a Proterozoic hematite.^13^ At the magnetite surface, the presence of surface U(V) has been observed under electrochemically controlled U(VI)-reducing conditions.^14,15^ Moreover, the nanoscale reductive mineralization mechanism has been uncovered, evidencing the formation of U oxide nanowires as an intermediate morphology, and the presence of U(V) as a transient valence state followed by reduction to U(IV).^16^ These results point to two consecutive one-electron transfers for the complete reduction of U(VI) on the magnetite surface.

U isotope composition (^238^U/^235^U) serves as a paleo-redox proxy to reconstruct the redox evolution of oceans and atmosphere throughout Earth’s history,^17−21^ and to monitor U transport or reductive remediation of U subsurface contamination in modern environments.^22−25^ Large variations of ^238^U/^235^U ratios were documented in natural U deposits and rocks, as well as in experimental studies, while the most significant uranium isotope fractionation occurs via the reduction of U(VI) to U(IV) in sediments that were deposited under anoxic/sulfidic conditions.^26^

Based on *ab initio* calculations,^27^ the equilibrium isotopic fractionation associated with the U(VI)–U(IV) redox transformation favors the accumulation of the heavy isotope (^238^U) in the U(IV) species, which is opposite to the direction expected for traditional mass-dependent stable isotope fractionation. Divergence from the typical direction of fractionation is attributed to an effect expected for heavy elements and associated with the interaction of the electron cloud with the nucleus, the nuclear field shift effect.^27−29^ Previous findings support the enrichment of ^238^U in reduction products in black shales,^30−32^ in bioremediated sediments,^24^ and during the reduction of U(VI) by microorganisms in the laboratory.^33−35^ In contrast, the isotopic fractionation of U during reduction by zerovalent iron (Fe^0^), aqueous Fe(II), or Fe(II)-bearing minerals exhibits three distinct behaviors: (a) no fractionation,^36,37^ (b) initially no preferential fractionation, followed by the preferential reduction of the light isotope (^235^U),^36^ or (c) preferential reduction of ^238^U, with a linear correlation between the percentage of neutral uranyl aqueous complexes and the isotope fractionation factor.^38^ The documentation of three distinct behaviors evidence unresolved questions about the controls on U isotopic fractionation during its reduction by Fe(II)-bearing minerals.

Because meaningful interpretation of δ^238^U data in the rock record and remediation sites depends on understanding U isotope systematics, the relationship between the mechanism of reduction and isotope fractionation must be unraveled. A recent study has investigated the uranium isotope fractionation associated with the coprecipitation of U(VI) and magnetite, revealing the light isotope associated with the mineral-incorporated U(V) species.^16^ Similar to U(V) as the intermediate valence state, pentavalent chromium [Cr(V)] exists as one of the intermediate valence states for Cr species, where the reduction of hexavalent Cr to Cr(V) by aqueous Fe(II) has been proposed as the rate-determining step^39^ and was found to contribute significantly to the overall Cr kinetic isotope fractionation.^40,41^ However, the isotope fractionation associated with the reduction of U(VI) to U(V) at an existing mineral surface (i.e., not coprecipitation) has not been investigated. As U(V) is an important intermediate in the reduction of U(VI) to U(IV),^14−16^ it may also impact the overall isotope fractionation behavior.

Therefore, the major objective of this study is to understand the role of the intermediate valence state U(V) in the overall isotope fractionation. The contribution of U(VI), U(V), and U(IV) was resolved by M_4_-edge high-energy-resolution fluorescence detection (HERFD) X-ray absorption near-edge structure (XANES), and the isotopic measurements were probed for U pools in either aqueous [U(VI)/U(V)], bicarbonate-extracted [U(VI)/U(V)], or solid phase [U(V)/U(IV)]. The observed variable U fractionation behavior in various U(VI)-magnetite systems was attributed to modulation of the contribution of U(V) to aqueous and bicarbonate-extracted phase U and to varying progress toward full reductive mineralization. This study provides insights into the impact of U(V) on the direction and magnitude of isotope fractionation during U(VI) reduction at the magnetite surface.

## Materials and Methods

### Sample Preparation

All experiments, including magnetite synthesis, reduction, and postreaction treatment, were performed in an anoxic chamber (MBRAUN) with an N_2_ atmosphere (O_2_ < 0.1 ppm). All reagents and chemicals were of ACS grade. Optima grade HCl was used for samples destined for isotope measurement. All aqueous solutions were prepared with Milli-Q water (18.2 MΩ cm) and deoxygenated by purging with N_2_ prior to transferring into the anoxic chamber. Solutions were equilibrated within the chamber for at least 24 h before usage. All serum bottles and butyl rubber septum were cleaned with analytical grade HCl, followed by Optima grade HCl and Milli-Q water prior to use. Synthesis of magnetite nanoparticles is described here^16^ and in the Supporting Information Text S1.

### U(VI) Reduction

Reduction experiments were performed with or without 20 mM piperazine-*N*,*N*′-bis(2-ethanesulfonic acid) (PIPES) buffer and with varied U(VI) and magnetite concentrations. PIPES/no-PIPES systems were compared in detail as preliminary investigations uncovered an effect of buffer on the isotopic fractionation behavior. Experimental conditions are listed in Table 1 and provided in Figure S1. The initial solution was prepared by amending a 20 mM natural uranium stock solution of the IRMM184 standard (Institute for Reference Materials and Measurements, IRMM) in a solution of 0.1 M HCl into serum bottles containing 1 or 2 mM NaHCO_3_ and either 20 mM PIPES (buffered at pH 7.0) or no PIPES. For batches with no PIPES buffer, the pH value of the suspension was adjusted to 7.0 ± 0.2 with 0.5 M HCl or NaOH, except for experiments 28-A and 28-B (pH ∼7.5) (Table 1). U(VI)-containing solutions were equilibrated inside the anoxic chamber for at least 2 h prior to addition of the magnetite suspension.

**Table 1:** Summary of Experimental Conditions^a^

magnetite stock no.	experiment name	Fe as Fe_3_O_4_ (mM)	U (μM)	Fe:U ratio [-]	PIPES (mM)	CO_3tot_ (mM)	samples for δ^238^U measurements^b^	solid phase characterization
stock 1	6.25-A	2.5	400	6.25	20	1	U-mag-bic-aq; *U-mag-bic- solid*	
	6.25-B	2.5	400	6.25	20	1	U-mag-bic-aq	
	12.5-A	5	400	12.5	20	1	U-mag-bic-aq; *U-mag-bic-solid*	
	12.5-B	5	400	12.5	20	1	U-mag-bic-aq	
	25-A	5	200	25	20	1	U-mag-bic-aq; *U-mag-bic-solid*	L_3_-edge XANES measurements for *U-mag* on 30 h
	25-B	5	200	25	20	1	U-mag-bic-aq; *U-mag-bic-solid*	
stock 2	25-PIPES	5	200	25	20	1	aqueous U; U-mag-bic-aq; *U-mag-bic-solid*	M_4_-edge XANES measurements for *U-mag* on 12, 24 h, 3 and 9 days for 25-PIPES and 25-noPIPES; TEM on 24 h *U-mag* samples for 25-PIPES and 25-noPIPES
	25-noPIPES	5	200	25		1	aqueous U; U-mag-bic-aq; *U-mag-bic-solid*	
stock 2	28-A	1.4	50	28		2^c^	U-mag-bic-aq	
	28-B	1.4	50	28		2^c^	U-mag-bic-aq	
stock 3	35.7-A	5	140	35.7	20	1	U-mag-bic-aq	
	35.7-B	5	140	35.7	20	1	U-mag-bic-aq	
stock 1	62.5-A	5	80	62.5	20	1	U-mag-bic-aq	L_3_-edge XANES measurements for *U-mag* on 30 h
	62.5-B	5	80	62.5	20	1	U-mag-bic-aq	
stock 3	62.5-C	5	80	62.5	20	1	U-mag-bic-aq	
	62.5-D	5	80	62.5	20	1	U-mag-bic-aq	
	62.5-E	5	80	62.5	20	1	U-mag-bic-aq; *U-mag-bic*-solid (sacrifice whole bottle)	

a Experiments are named based on the molar Fe to U ratio (e.g., 2.5 mM Fe and 400 μM U yield 6.25).

b *U-mag* represents the magnetite-associated U in the solid; “U-mag-bic-aq” represents the extracted aqueous phase after extraction with a bicarbonate solution; “*U-mag-bic-solid*” represents the remaining solid phase after bicarbonate extraction; and “Aqueous U” represents the aqueous phase U in the solution.

c The pH value of all experiments was controlled at pH 7, except for ^c^, where 2 mM NaHCO_3_ was used to have better pH buffer capacity during the Exp. 28-A and 28-B, and the final pH was ∼7.5.

For experiments 25-PIPES and no-PIPES, aliquots (>2 mL) were withdrawn at the desired time intervals, with supernatants being separated from the solid phase by a strong magnet and filtered through 0.22 μm PTFE filters (ThermoFisher, USA) to quantify the dissolved U species in the filtrate (abbreviated aqueous U) (Figure S1). After magnet separation, the magnetite-associated U in the solid phase (*U-mag*) was collected as a wet paste to quantify U speciation by X-ray absorption spectroscopy (XAS) measurements. In addition, the magnetite suspension was mixed with an equal volume of 200 mM anoxic NaHCO_3,_ resulting in 100 mM bicarbonate, for a 30 min extraction, which was determined to be sufficiently long to extract unreduced U(VI) and extractable U(V) into the solution.^16^ Meanwhile, the short contact time would preclude the possibility of isotope exchange between U in different pools [such as extracted U(VI) and solid phase U(IV)^42^]. The solid phase (containing unextractable U) was separated with a magnet, and the supernatant was filtered through 0.22 μm PTFE filters and analyzed for U concentration and isotopic fractionation. U obtained from the extracted supernatant (U-mag-bic-aq) was interpreted as unreduced uranyl(VI) species that desorbed from the magnetite surface by forming soluble uranyl-carbonate complexes.^43^ The U associated with the solid phase after bicarbonate extraction (*U-mag-bic-solid)* and collected after magnet separation was digested in 3 N Optima grade HCl to monitor unextractable solid-associated U concentrations and δ^238^U values.

For all other experiments, filtration (0.22 μm PTFE) was applied (with no magnet separation) to obtain the filtered solids, and in addition, the solids from 25-A and 62.5-A were collected for XAS measurements. Additionally, after mixing with a bicarbonate solution, suspensions were filtered again, and the filtered solids and filtrates were collected separately as *U-mag-bic-solid* and U-mag-bic-aq (Figure S1).

### Aqueous Phase and Isotope Analysis

Solutions resulting from bicarbonate extraction or from the digestion of the solid phase were diluted in 1% HNO_3_, and the U concentration was measured by inductively coupled plasma-mass spectrometry (ICP-MS, PerkinElmer or Agilent 7900). Undiluted samples were stored and shipped to the Leibniz University Hannover (Germany) for isotope measurements. Detailed procedures for isotope measurements using a ThermoScientific-Neptune multicollector inductively coupled plasma source mass spectrometer (MC-ICP-MS) were described in our previous study.^16^ Briefly, samples were pretreated and then purified by the ion-exchange chromatographic method with the Eichrom UTEVA resin.^30^ Prior to resin separation, samples were dissolved in 3 M HNO_3_ and spiked with a weighted aliquot of the ^236^U/^233^U isotope tracer (IRMM 3636-A, ^236^U/^233^U = 0.98130) to correct for potential isotope fractionation during U separation and instrumental mass discrimination during isotope measurements. Triplicate analyses were performed for each sample, and the precision was reported as two standard deviations (2 S.D.) of the triplicate analyses, which is typically ≤0.1‰.

### Solid Phase Characterization

The U-containing magnetite solid phases were characterized by M_4_-edge HERFD-XANES spectroscopy and L_3_-edge XANES to determine the U valence state. Detailed sample preparation and measurement setup are described in Text S2. Acquired spectra of samples were interpreted by linear combination fitting using Athena^44^ or iterative-target transformation factor analysis (ITFA)^45^ to quantify the contribution of the three different U valence states.^9,16^ Magnetite was imaged by transmission electron microscopy (TEM), sample preparation is described in Text S1, and example micrographs are shown in Figure S2.

## Results

### Aqueous Phase Analysis

**Figure 1:**
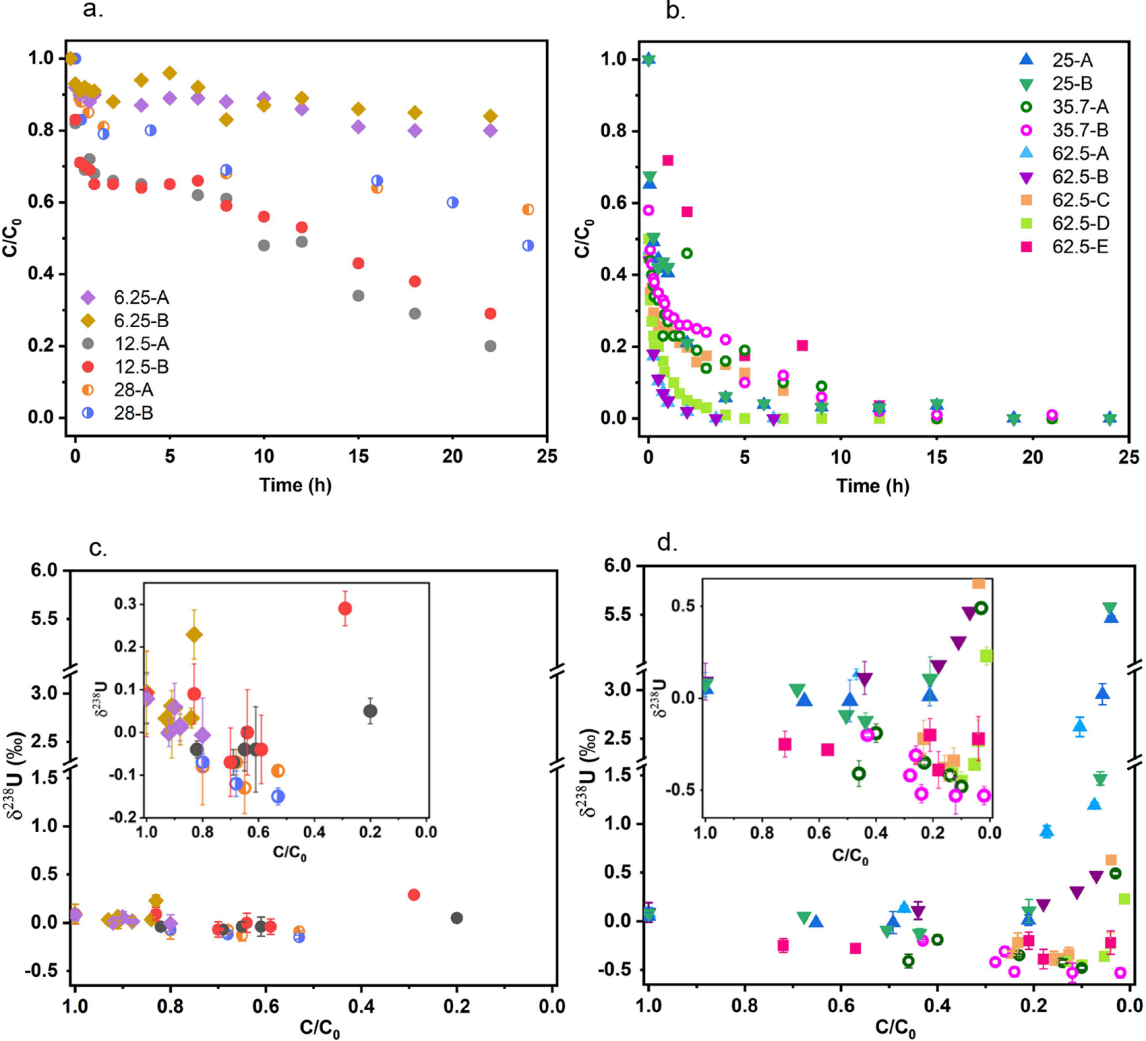
(a, b) Time-dependent bicarbonate-extracted uranium concentration for U(VI) incubated with magnetite at varying Fe:U ratios (Table 1) and (c, d) the corresponding δ^238^U (‰) of bicarbonate-extracted U (U-mag-bic-aq). (a) and (c): batches with slow reduction kinetics (*t*_1/2_ ≥ 12 h); (b) and (d): batches with rapid reduction kinetics (*t*_1/2_ < 12 h). *t*_1/2_ represents the reaction time to reach *C*/*C*_0_ of 0.5. *C*/*C*_0_ represents the ratio of the concentration of U that could be extracted by a bicarbonate solution to the initial uranium concentration. At least replicate experiments (A and B) were performed for each Fe:U ratio, and data for each reaction batch are presented. The Fe:U is indicated for each experiment. The inset in (c) and (d) represents δ^238^U values with a zoomed-in *y*-axis scale. A small contribution of aqueous phase U was also included in the bicarbonate-extracted phase. Error bars in (c) and (d) represent isotopic measurement 2 SD values. The concentration (*C*) in *C*/*C*_0_ always refers to bicarbonate-extractable U.

During U(VI) reduction by magnetite nanoparticles, U was removed rapidly from the aqueous phase (Figure S3a),^16^ while concentrations of bicarbonate-extracted U (U-mag-bic-aq) decreased gradually over time under all experimental conditions (Figures 1a,b, and S3b). Bicarbonate-extracted U is intended to represent U(VI) remaining on the magnetite surface; however, as discussed in later sections, some U(V) is also extracted. Therefore, the decreasing concentrations indicate the progressive reduction of U(VI) and extractable U(V) to unextractable U(V) and U(IV). The reduction kinetics, based on the changing concentrations of bicarbonate-extractable U, varied among the experiments. The surface properties of magnetite, such as the surface area, U loading, aging of magnetite stocks, and surface Fe(II)/Fe(III) ratio, likely all contribute to the varied reduction kinetics.^2−4^ Therefore, the required reaction time to reach *C*/*C*_0_ of 0.5 (*t*_1/2_) was compared to categorize the experiments into fast (*t*_1/2_ < 12 h) or slow (*t*_1/2_ ≥ 12 h) reaction groups (Figure 1a,b). The slowest reduction was observed at the lowest Fe:U ratio of 6.25 (2.5 mM Fe and 400 μM U), with 80% unreacted U(VI)) after a 24 h reaction (Figure 1a). Meanwhile, the most rapid reduction occurred at the highest Fe:U ratio of 62.5 (5 mM Fe and 80 μM U, 62.5-A, -B, and -D), where less than 20% of U was bicarbonate-extractable after 5 h (Figure 1b).

Additionally, in parallel experiments that included or excluded PIPES buffer (25-PIPES and 25-noPIPES), the concentration of both aqueous U and bicarbonate-extracted U (U-mag-bic-aq) was higher for the PIPES than for the no-PIPES treatment at the same sampling times, indicating that, in the presence of PIPES, the reduction of surface-adsorbed U might be slower than that in its absence (Figure S3a,b).

### Solid Phase Characterization

Either L_3_-edge XANES or M_4_-edge HERFD-XANES spectroscopy was used to confirm the extent of U reduction and to characterize the composition of U valence states in reduction products from select experiments. By comparing the L_3_-edge XANES spectra of magnetite-associated U in the solid-phase samples obtained at 30 h from 25 to 62.5-A by direct filtration (Figure S4), we observed the accumulation of U(IV) and U(V) from the reduction of U(VI) as a function of time. Additionally, in the experiment comparing the PIPES and no-PIPES treatments (25-PIPES and 25-noPIPES), the U valence composition in the solid phase was determined by ITFA analysis of the M_4_-edge HERFD-XANES data as a function of the reaction progress (Figures 3 and S5). Early in the reaction, up until about *C*/*C*_0_∼0.35, the two conditions exhibit similar U solid phase speciation, with U(V) representing the dominant species (∼49–56%) and the PIPES condition showing a lesser extent of reduction than no PIPES [more U(V) and less U(IV)] while the fraction of U(VI) is almost identical. However, as the reaction reaches *C*/*C*_0_ ∼ 0.08 (representing 24 h for no-PIPES and 3 days for PIPES) and ∼14–18 μM U extractable, the solid phase speciation is distinct. The PIPES system includes 44% U(IV) and 50% U(V), whereas the no-PIPES system exhibits 30% U(IV) and 55% U(V). Therefore, at similar extractable U (*C*/*C*_0_), the solid phase is more reduced in the presence of PIPES than in its absence (Figure 3).

### Isotope Measurements

The δ^238^U values of the bicarbonate-extracted phase exhibited two distinct trends of isotope fractionation (Figure 1c,d): (a) A trend of decreasing δ^238^U with decreasing *C*/*C*_0_ observed for rapid reactions starting at *C*/*C*_0_ ∼0.5 (e.g., 35.7-A, 35.7-B, 62.5-C for which δ^238^U values decrease from −0.19 to −0.48‰, −0.2 to −0.53‰, and −0.22 to −0.34‰ for *C*/*C*_0_ from 0.40 to 0.10, 0.43 to 0.02, and 0.127, respectively), and to a lesser extent for slower reactions for *C*/*C*_0_ between 1.0 to 0.5 (e.g., 6.25-A, 12.5-B, and 28-B for which δ^238^U values decrease from 0.08 to −0.01‰, 0.09 to −0.04‰, and −0.07 to −0.15‰, respectively). (b) A second group of experiments (62.5-A, 62.5-B, 62.5-C, 25-A, and 25-B) showing a steep increase in δ^238^U with decreasing *C*/*C*_0_ (for *C*/*C*_0_ < 0.1) up to values of ∼ +5‰.

**Figure 2:**
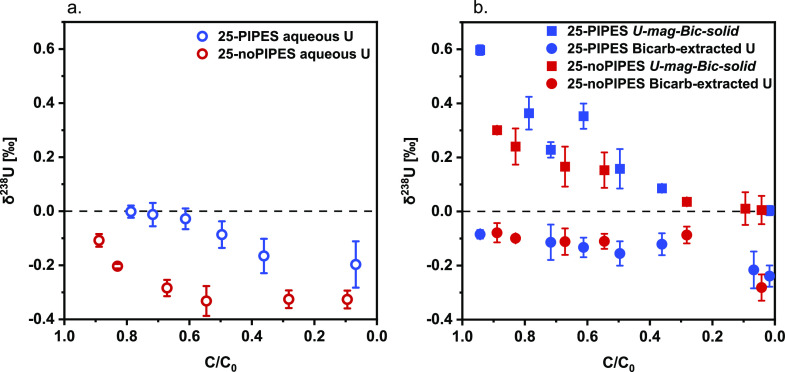
U(VI) reduction by magnetite in the presence and absence of 20 mM PIPES (25-PIPES and 25-noPIPES). The δ^238^U values (‰) of aqueous U (open circles) (a), bicarbonate-extracted U (U-mag-bic-aq, filled circles) (b), and the remaining U in solid (*U-mag-bic-solid*, filled squares) (b) for the system with PIPES or without PIPES (no PIPES). Both experiments were performed with 200 μM U and 5 mM Fe and only differed due to the presence/absence of 20 mM PIPES. *C*/*C*_0_ represents the ratio of the concentration of U that could be extracted by a bicarbonate solution to the initial uranium concentration. Error bars in (a) and (b) represent isotopic measurement 2 SD values. The concentration (*C*/*C*_0_) of time-dependent aqueous U and bicarbonate-extracted U is included in Figure S3. A small contribution of aqueous phase U was also included in the bicarbonate-extracted phase. The isotope measurements for a replicate experiment with or without PIPES are also included in the Supporting Information. The concentration (*C*) in *C*/*C*_0_ always refers to bicarbonate-extractable U.

In addition to bicarbonate-extracted U, aqueous U samples were collected for isotope measurements for PIPES/no-PIPES comparison. The aqueous δ^238^U values were substantially different for PIPES and no-PIPES. While the PIPES condition exhibits δ^238^U values decreasing gradually from 0 to −0.2‰, the no-PIPES system shows a steep decrease in δ^238^U values for *C*/*C*_0_ from 0.9 to 0.55 (from −0.1 to −0.33‰) and little change in the value subsequently (Figure 2a). In contrast, the δ^238^U values of bicarbonate-extracted U are similar for both conditions (Figure 2b). In both cases, the δ^238^U values remain constant at ∼ −0.1‰ up until *C*/*C*_0_ ∼ 0.3 and then decrease to ∼ −0.25‰ as the reaction progresses.

Additionally, the difference between the aqueous and bicarbonate-extracted δ^238^U values varies between the PIPES and no-PIPES systems (Figure S3c,d, replicate experiment in Figure S6). For the no-PIPES system, the aqueous δ^238^U values for both aqueous and bicarbonate-extracted U exhibit a negative value at the first measured *C*/*C*_0_ of 0.88 with δ^238^U for aqueous U being slightly more negative than that for bicarbonate-extracted U. The offset grows larger over the course of the reaction up until a *C*/*C*_0_ of 0.28. In contrast, in the PIPES system, the δ^238^U values for bicarbonate-extracted U were slightly more negative than the aqueous values system, with an offset of about 0.1‰ at *C*/*C*_0_ ∼0.7 that disappeared as the reaction progressed.

The negative δ^238^U values observed for aqueous or bicarbonate-extracted U for most time points (Figure 1c,d) strongly indicate the accumulation of the heavy isotope into the solid U species. This was confirmed by the determination of the U isotopic composition of the unextractable solid-associated U (i.e., that remaining after extraction) for a subset of experiments (Figure 4). Interestingly, regardless of the range of reduction kinetics and δ^238^U values in the aqueous or the bicarbonate-extracted samples, a similar trend of δ^238^U was observed in all unextractable solid-associated U (*U-mag-bic-solid*). In Exp. 25-A and 25-B, the δ^238^U of solid U were slightly higher (reached up to 0.1‰) than others (∼0‰) near *C*/*C*_0_ ∼ 0 (Figure 4). For the rest of the experiments, the δ^238^U was high (∼0.5‰) initially (at a *C*/*C*_0_ of ∼0.9) and decreased over time to reach 0‰ when the reaction neared completion (*C*/*C*_0_ ∼ 0).

## Discussion

### U(V) Persistence during U(VI) Reduction by Magnetite

**Figure 3:**
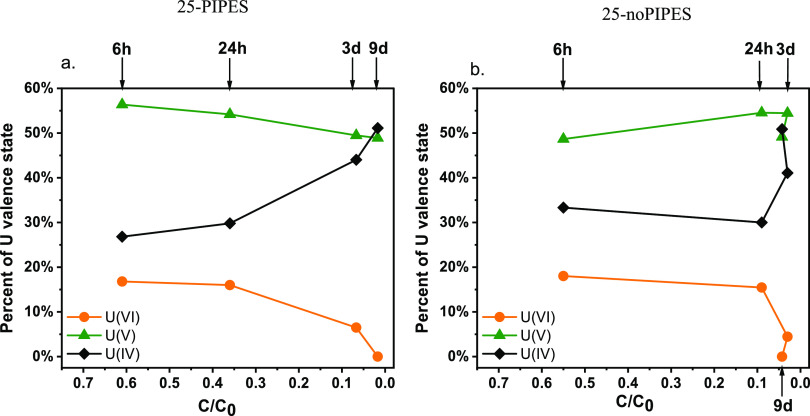
Composition of U valence states from M_4_-edge HERFD-XANES measurements of *U-mag* solid phase for experiments (a) with PIPES (25-PIPES) and (b) without PIPES (25-noPIPES). Samples were collected at 6, 24 h, 3 and 9 days. Both (a) and (b) were obtained from the reaction of 200 μM U with 5 mM Fe. *C*/*C*_0_ represents the ratio of the concentration of U that was obtained by a bicarbonate solution relative to the initial uranium concentration. The M_4_-edge HERFD-XANES spectra are presented in Figure S5. The corresponding estimated root-mean-square (RMS) error associated with the ITFA analysis^9^ was reported as 1% for U(IV) and 2% for U(V) and U(VI), representing the relative error. The concentration (*C*) in *C*/*C*_0_ always refers to bicarbonate-extractable U.

Experimental evidence for U(V) persistence in iron oxides has been accumulating for more than 10 years.^8^ The most recent work has evidenced the presence of U(V) in a 1.6 billion years old hematite.^13^ Previous work has both reported the persistence of U(V) up to 4 days as well as the formation of transient nanowires composed of individual uranium oxide nanoparticles that later collapse into UO_2_ nanoclusters.^16^ U(V) persistence is observed for the systems characterized by XAS in this work (Figures 3, S4, and S5). Indeed, U(V) is a major contributing valence state after 30 h in one case (Figure S4) and up to 9 days in the other (Figure 3). Furthermore, we observe nanowires with the same morphology as previously reported (Figure S7). Thus, in contrast to previous interpretations of U isotope fractionation,^36,38^ here, we explicitly consider the role of U(V) during U(VI) reduction.

We posit that the puzzling observation that U isotope fractionation varies depending on Fe:U (Figure 1) can be partly explained by the variation in U(V) concentration across the conditions. Considering the evidence that U(V) is present at the magnetite surface, the assumption that bicarbonate extraction only recovers U(VI) must be re-evaluated. Indeed, the data presented in Figure 1, showing a wide range of δ^238^U values at *C*/*C*_0_ ∼ 0, all stem from bicarbonate-extracted samples. Thus, we hypothesize that bicarbonate extraction may target both U(VI) and a fraction of labile U(V). Evidence for this hypothesis is provided by comparing U-M_4_-edge HERFD XANES and bicarbonate extraction results (Figures S3b and 3). After 6 h, the solid phase (in the presence of PIPES) includes 17% U(VI), 56% U(V), and 27% U(IV), based on spectroscopic analysis (Figure 3a). In contrast, the same sample extracted with 100 mM bicarbonate exhibits 60% extractable U (Figure S3b). This suggests that, in addition to U(VI), 76% of the U(V) is also extractable. Similarly, at 24 h, 36% of U was bicarbonate-extractable, while U(VI) represented only 16% of total U. This means that ∼37% of the total U(V) is extractable by bicarbonate at 24 h. At 3 days, the extractable U represents 7%, while U(VI) represents 6.5%, evidencing a convergence between the values of the two types of measurements. Similar results were observed in our previous study with 5 mM Fe and 200 μM U (in the presence of PIPES): although ∼24% of U in *U-mag* solid was detected as U(VI), ∼35% of total U was bicarbonate-extractable.^16^ This observation also holds for the no-PIPES system: after 6 h, ∼18% of U was detected as U(VI) via XANES (Figure 3b), whereas 55% was bicarbonate-extractable (Figure S3b). This represents about 76% of U(V) as bicarbonate-extractable. We observe that, in general, as the reaction progresses, the discrepancy between the XANES-derived fraction of U(VI) and the fraction extracted by bicarbonate decreases, suggesting that U(V) is progressively transformed into a less labile (less bicarbonate-extractable) form. However, in contrast to the PIPES system, the convergence in values of U(VI) by XANES and by bicarbonate extraction in the no-PIPES system is reached sooner (at 24 h), suggesting that the transformation of U(V) to an unextractable form was faster in the absence of PIPES. Thus, the decreasing amount of bicarbonate-extracted U in the current study suggests not only the reduction of U(VI) to U(V) but also the transformation of U(V) species from bicarbonate-extractable to -unextractable phases, which would largely depend on the reduction progress and the associated U oxide morphology and/or coordination environment.

A previous study synthesized UO_2_(CO_3_)_3_^5–^ as a stable U(V)-carbonato complex in an aqueous phase under high carbonate concentration (1 M) and high pH (>11).^46^ With a circumneutral pH value and relatively low carbonate concentration (1 to 2 mM carbonate), the formation of UO_2_(CO_3_)_3_^5–^ species in the aqueous phase is unlikely in the present study. As understanding of the mechanism by which U(V) is mobilized by the bicarbonate extraction or of the atomic-scale transformation of U(V) to uraninite (UO_2_) nanoparticles remains poor, it is currently not possible to predict the change in extractable U(V) concentration over time.

### U Isotope Fractionation Behavior

Inconsistencies in the U isotope fractionation behavior during reduction by reduced Fe-bearing minerals with a wide range of reduction kinetics currently lack an explanation. Consideration of U(V) species may be one of the keys to unraveling the variation in the U(VI) isotope fractionation behavior during its reduction by ferrous iron-bearing minerals. For instance, Stylo’s study^36^ evidenced rapid U(VI) reduction by magnetite based on the change in bicarbonate-extracted U concentrations, and no isotope fractionation was observed in the early phases of the reduction, followed by the preferential reduction of the light isotope. Similar fractionation behavior was observed in 25-A, 25-B, and 62.5-A in the current study. Brown’s work on U(VI) reduction by iron monosulfide showed the preferential accumulation of the light isotope in aqueous phase U.^38^ Neither of the two studies^36,38^ considered the presence of U(V) as an intermediate reduction product, while we and others have evidenced its presence during U(VI) reduction by magnetite^16^ and iron sulfide.^47^

We first interpret the isotopic fractionation obtained for bicarbonate-extracted U for the PIPES/no-PIPES systems (Figure 2b). The two data sets are very similar. Indeed, we observe that the δ^238^U values are stable at around 0.1‰ up until *C*/*C*_0_ reaches a value of ∼0.3, at which point, the isotopic signature of extractable U becomes more negative, down to ∼ −0.23 or −0.28‰ at *C*/*C*_0_ 0.017 or 0.042 for PIPES and no-PIPES, respectively. *Ab initio* calculations show that U(V) species exhibit a positive equilibrium isotopic fractionation relative to U(VI).^48^ Thus, the biphasic behavior of the δ^238^U values as a function of *C*/*C*_0_ is reasonably attributable to the combined signature of U(VI) and U(V) in extracted U, with U(VI) light and U(V) heavy, followed by the decreasing contribution of U(V) as it becomes increasingly less extractable. The presence of PIPES appears to impact the rate of conversion of extractable to unextractable U(V).

In contrast, there was a large difference between the δ^238^U values of aqueous U between the PIPES and the no-PIPES systems (Figure 2a). It is important to note that while δ^238^U values exhibit different values, the amount of U represented by these pools is minimal (≤2% of the total U). Nonetheless, the heavier δ^238^U values in the PIPES system suggest stabilization in solution of low concentrations of U(V), likely by PIPES, resulting in slightly heavier δ^238^U values. Therefore, while there is strong evidence indicating the presence of bicarbonate-mediated labile U(V), its presence in aqueous (unextracted) samples must also be considered.

In the PIPES system, only slight differences were observed between δ^238^U values for aqueous and bicarbonate-extracted U, with the δ^238^U values for bicarbonate-extracted U slightly lighter at the beginning of the reduction (Figure S3c). This observation can be explained by bicarbonate-extracted U containing a higher amount of light U(VI) extracted from the surface, especially at the beginning of the reduction process, leading to more negative δ^238^U values for bicarbonate-extracted U than for aqueous U.

However, in the no-PIPES treatment, we propose that aqueous U (<0.22 μM after 6 h, corresponding to bicarbonate-extractable U at *C*/*C*_0_ = 0.55) contains mostly U(VI) (δ^238^U < 0), with low or no U(V) (δ^238^U > 0), leading to negative δ^238^U values from the first measurement (−0.1‰ at *C*/*C*_0_ = 0.88). In the bicarbonate-extracted phase, the contribution of bicarbonate-extractable U(V) (δ^238^U > 0) results in heavier δ^238^U values than for aqueous U. However, after 24 h of reaction (*C*/*C*_0_ < 0.09), when U(V) could no longer be extracted, the difference in δ^238^U values between the aqueous and bicarbonate-extracted U disappeared. In summary, to explain the PIPES/no-PIPES data, we must invoke (a) the stabilization of U(V) in solution by PIPES and (b) a bicarbonate-extractable surface U(V) fraction that decreases over time.

**Figure 4:**
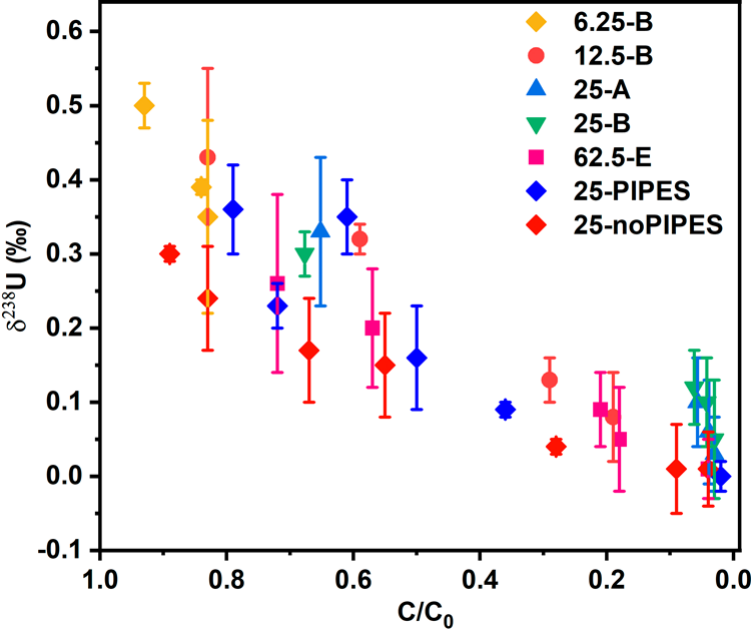
δ^238^U values (‰) of the remaining U in solid (*U-mag-bic-solid*) after bicarbonate extraction. *C*/*C*_0_ = 1 represents the beginning of the reduction experiments, while *C*/*C*_0_ = 0 represents that U could no longer be extracted by a bicarbonate solution. For Exp. 62.5-E, reactors at each time point were sacrificed for isotopic measurements. For Exp. 6.25-B, 12.5-B, 25-A, and 25-B, 500 μL aliquot was withdrawn and incubated in an equal volume of 200 μM anoxic NaHCO_3_ for 12 h, and the suspension was filtered by 0.22 μm PTFE filters, which were acid-washed to measure δ^238^U in *U-mag-bic-solid*. For 25-PIPES and 25-noPIPES, at least 2 mL aliquot was withdrawn from the batch and incubated in an equal volume of 200 μM anoxic NaHCO_3_ for 30 min. Afterward, a strong magnet was applied first to collect the *U-mag-bic-solid,* while the supernatants were filtered to collect U-mag-bic-aq. The solid phase data for 25-PIPES and 25-noPIPES are also presented in Figure 2. Error bars represent isotopic measurement 2 SD values. The concentration (*C*) in *C*/*C*_0_ always refers to bicarbonate-extractable U.

The detailed investigation and comparison of the PIPES/no PIPES systems establish a framework with which to interpret the data from other experiments (Figure 1). The positive δ^238^U values for unextractable solid-associated U after bicarbonate extraction (*U-mag-bic-solid*), most likely U(IV) oxide or U(V)/U(IV) mixed valence U oxides, indicate that reduction products carry a heavy isotope composition (Figure 4), and thus, the light isotope should accumulate in the U(VI) species.

For most experiments, biphasic behavior was observed. For slow reductions, most δ^238^U values of the bicarbonate-extracted U decrease from 0.1 to −0.15‰ early in the reaction, within the range of *C*/*C*_0_ = 1 to 0.5 (there are few measurements beyond that point) (Figure 1c). In contrast, for fast reductions (Figure 1d), there is little change in δ^238^U up until values of *C*/*C*_0_ of around 0.5. Beyond that point, we observe either a decrease of δ^238^U down to −0.5‰ with more scatter in the values than for slow reductions or a steep increase for *C*/*C*_0_ ≤ 0.5 up to values of >5‰, representing values an order of magnitude larger (Figure 1d). We interpret the first two biphasic behaviors as being related to U(V) formation. During the first half of the reaction (*C*/*C*_0_ from 1 to 0.5) for the slow kinetics cases, an increasing amount of U(V) is formed, and as the bicarbonate-extracted U consists of both U(VI) and U(V), the presence of both light U(VI) and slightly heavy U(V) in the extracted U pool results in measured δ^238^U values close to 0. For the fast kinetics cases, from *C*/*C*_0_ = 0.5 on, the continued reduction of U(VI) to U(V) and U(IV) results in a decreasing amount of surface-associated U(VI) and, depending on how rapidly U(V) is reduced, to a larger contribution of slightly heavy U(V). As the δ^238^U values for the bicarbonate-extracted solution are contingent on the relative contributions of U(VI) and bicarbonate-extractable U(V), it is expected that they would vary with the fraction of U(V) extracted. Therefore, we interpret most of the results for which δ^238^U values increase (e.g., 62.5-B, 62.5-C, and 35.7-A) as a greater contribution of U(V). Naturally, the ratio of bicarbonate-extractable U(V) to the total amount of U(V) depends on U(V) speciation. We hypothesize that bicarbonate extraction targets only some of the U(V)-bearing U oxides. Therefore, depending on U(V) speciation, the extraction may result in varying amounts of U(V) extracted, contributing to the variable overall isotope compositions across experimental conditions.

However, the contribution of U(V) is not able to explain the extremely high δ^238^U values of bicarbonate-extracted U observed in experiments 25-A, 25-B, and 62.5-A after *C*/*C*_0_ < 0.2. Similar isotopic effects were already observed by Stylo et al. for U(VI) reduction by magnetite and aqueous Fe(II).^36^ There are several mechanisms that could explain this observation: (a) As kinetic theory indicates that for a multistep reaction, the steps up until and including the rate-limiting step determine the isotope fractionation. Therefore, if the rate-limiting step involves the creation of weaker bonds and occurs prior to electron transfer, then mass-dependent fractionation may be a dominant contributor to the overall signal. For instance, in this case, if the rate-limiting step is the rearrangement and lengthening of bonds in uranium oxides following the reduction to U(IV), isotopic fractionation may produce very heavy remaining U(VI); (b) This observation may be explained by a switch from a bonding-related to a surface reaction kinetic isotope fractionation mechanism that may occur when the amount of remaining oxidized uranium is small.^49^ Such a scenario would result in a strong enrichment of the heavy ^238^U in the remaining U(VI), i.e., in the bicarbonate-extracted phase, because of the faster reaction rate of light isotopes (^235^U). This regime was described for calcite precipitation as corresponding to the precipitation rate exceeding the rate of forward or backward reaction.^49^ We invoke the same mechanism by which the reaction rate is greater than the supply of the reagent to the surface. In that case, the lighter isotope is transported more quickly, resulting in a heavy isotope composition in the remaining U(VI). Thus, it is not surprising that this behavior is observed only within the most rapid reactions.

Interestingly, the δ^238^U values of unextractable solid-associated U (after bicarbonate extraction) all show positive δ^238^U values at the beginning of the reaction followed by a continuous decrease until nearing 0‰ at *C*/*C*_0_ = 0 (Figure 4). At the beginning of the reduction process, U is mostly adsorbed as U(VI) on the magnetite surface. Thus, the initial heavy δ^238^U observed in the unextractable solid phase U reflects fractionation of the small amount of unextractable U(V) or U(IV). As the reduction proceeds, the reactant pool becomes progressively lighter and produces lighter products, leading to the decreasing δ^238^U values in the unextractable solid-associated U and final δ^238^U values close to 0. Overall U isotope fractionation behavior varied across reactions with fast and slow reduction rates, with some displaying significant kinetic effects while others did not. We posit that the isotope fractionation behavior across various experimental conditions depended largely on the reduction mechanism, in other words, the reduction kinetics of the U(VI) to U(V) step vs the U(V) to U(IV) step, and the reactivity of the uranium oxide species in which U(V) is likely sequestered.

For reactions where both bicarbonate-extracted solutions and solids were collected, isotopic mass balance was achieved for most cases except in those experiments in which unusually small samples were collected for isotopic measurement (i.e., 25-A, 25-B, 6.25-B, and 12.5-B) that resulted in overall δ^238^U values higher than 0‰ (Figure S8). Detailed discussions on the isotopic mass balance are shown in the Supporting Information Text S3.

### Environmental Implications

Due to its redox sensitivity and long residence time in the ocean (∼500 ky), U is considered as a reliable paleo-redox tracer to reconstruct Earth’s past atmosphere and oceans^17−21^ as well as a monitoring tool to trace U(VI) reduction in the modern environments.^22−25^ While the current understanding is grounded in the isotope fractionation that occurs during the transition from U(VI) to U(IV), the existence of U(V) and its role in the fractionation processes have been largely overlooked. In light of the identification of U(V) in 1.6 billion years old hematite,^13^ it is increasingly clear that U isotope fractionation during Fe(II)-mediated reduction must consider intermediate valence states and their attendant fractionation behavior.

U isotopic fractionation during microbial reduction presents a range of values that have been related to electron flux from the cell to U,^50^ but the signatures consistently recapitulate the dominance of the nuclear field shift effect, with the heavy isotope preferentially accumulating in the reduced phase. In contrast, in abiotic systems, U isotope fractionation has been reported in both directions. Here, we provide support for the hypothesis that the direction of fractionation is controlled by a reduction mechanism. The findings of the current study point to the presence of several U(V) solid-phase and aqueous species as key to explaining the variability of δ^238^U values among abiotic studies and to unraveling the inconsistent behavior across systems. The results also raise the possibility that, in rapid reactions, very high and positive fractionation values are obtained as a result of preferential transport of the light isotope or bonding re-arrangement of uranium species.

The identification of mobile U(V) species bearing heavy isotopes could improve the interpretation of δ^238^U values to decipher the reduction extent. Large variability in δ^238^U values has been observed in iron-rich rocks from 2.95 Ga on. The negative isotope accumulations comparing to igneous rocks were interpreted as due to the adsorption of dissolved U(VI) or the reduction of U(VI) by Fe(II) to U(IV) species.^32^ The large variation of δ^238^U values might be the result of multistep reactions of U(VI) to U(V) and U(V) to U(IV) with varied reduction kinetics. In addition, U(VI) may be sequestered into carbonate rocks during their formation, and they are thought to capture seawater U δ^238^U values. Thus, the extremely heavy U(VI) observed under low remaining oxidized U concentrations and resulting from either bonding-related or surface reaction kinetic effects may account for heavier than seawater U observed in carbonate rocks in both paleo and modern environments.^51,52^ Yet, this newfound understanding also presents challenges in applying U isotopes as a paleo-redox proxy. Additional work utilizing electrochemical reduction to isolate individual one-electron transfer processes would be necessary to further understand the role of U(V) in U isotope fractionation behavior. Moreover, a better understanding of the atomic-scale mineralization process associated with the two-step electron transfer, as well as of the persistence and morphology transformation of U(V), will be required to thoroughly constrain the U isotope fractionation behavior.

## Data Availability

All relevant data are available from the authors or within the Supporting Information files. Raw data, including isotope information for each measurement, are available from a data repository as https://doi.org/10.5281/zenodo.10894108.

## Supporting Information Available

Synthesis of magnetite; L_3_-edge XANES and M4-edge HERFD-XANES measurement and spectra; discussion on isotope mass balance of experiments where both bicarbonate-extracted solutions and the remaining solids measured for isotope composition; and HAADF-STEM images of U-magnetite samples (PDF)
