# Supplementary material for: Pentavalent U Reactivity Impacts U Isotopic Fractionation during Reduction by Magnetite
Source: Environ Sci Technol. 2024 Apr 4;58(15):6595–604. doi: 10.1021/acs.est.3c10324 (PMC11025122; doi:10.1021/acs.est.3c10324)
Supplement: Supplementary file 1 — es3c10324_si_001.pdf [file es3c10324_si_001.pdf]

Supporting Information

# Pentavalent U reactivity impacts U isotopic fractionation during reduction by magnetite

*Zezhen Pan*[1,2,3], *Luca Loreggian*[2], *Yvonne Roebbert*[4], *Barbora Bartova*[2], *Myrtille O.J.Y. Hunault*[5], *Stefan Weyer*[4], *Rizlan Bernier-Latmani*[2,*]

[1] Department of Environmental Science and Engineering, Fudan University, Shanghai 200438, China

[2] EML, École polytechnique fédérale de Lausanne, Lausanne, 1015, Switzerland

[3] Institute of Eco-Chongming (IEC), Shanghai 200062, China

[4] Synchrotron SOLEIL, L'Orme des Merisiers, Saint Aubin BP 48, 91192 Gif-sur-Yvette, France

[5] Institut für Mineralogie, Leibniz Universität Hannover, D-30167 Hannover, Germany

Submission to *Environmental Science & Technology*

This Supporting Information contains 13 pages, 3 sessions of text and 8 figures.

SI content:

| | |
|---|---|
| Text S1 | Magnetite synthesis |
| Text S2 | $L_3$-edge XANES and M4-edge HERFD XANES measurement |
| Text S3 | Isotope mass balance |
| Figure S1 | Schematic of experimental manipulations |
| Figure S2 | TEM images of magnetite nanoparticles (before reaction) |
| Figure S3 | The concentration ($C/C_0$) of time-dependent aqueous U and bicarbonate extracted U and the corresponding $\delta^{238}U$ values in PIPES and no-PIPES systems |
| Figure S4 | Normalized bulk U $L_3$ edge XANES spectra of *U-mag* samples |
| Figure S5 | $M_4$-edge HERFD-XANES spectra of Exp. 25-PIPES and 25-noPIPES |
| Figure S6 | Isotopic measurement for aqueous and bicarbonate extracted U from replicate experiment of 25-PIPES and 25-noPIPES |
| Figure S7 | HAADF-SEM images for *U-mag* samples |
| Figure S8 | Isotope mass balance overview |

*Text S1. Magnetite synthesis and Transmission electron microscopy imaging.* $Fe_3O_4$ nanoparticles were synthesized via a co-precipitation method, modified from Wang et al.[1] and was employed in our previous study[2]. In a nitrogen-filled anoxic chamber (MBRAUN), a mixture of 40 mL of 1 mol.L$^{-1}$ $FeCl_3$ and 20 mL of 1 mol.L$^{-1}$ $FeCl_2$ was continuously stirred in a glass beaker, and the pH of the mixture was increased by steadily adding 1 M NaOH dropwise until a pH value of ~11 was reached and maintained for at least half an hour. Post-synthesis suspensions were transferred to a clean serum bottle, settled overnight and checked again for pH, which was re-adjusted back to 11 if needed. The solid was separated from the suspension by an Nd magnet and washed twice with Milli-Q water to remove salts. The solid was resuspended in MilliQ water and sealed in serum bottle with a butyl rubber septum for further use.

Magnetite nanoparticles or U-associated magnetite nanoparticles were imaged by transmission electron microscopy (TEM) following the preparation procedures reported in our previous study[2]. Briefly, the solids were separated from the suspensions (10-50 μL) by an Nd magnet followed by decantation of the liquid, then the collected solids were re-dispersed into 1 mL 70% ethanol (oxygen-free), sealed in vials and sonicated for 3 mins. Then 2-5 droplets of the re-dispersed suspensions were deposited onto lacey carbon grids or ultra-thin carbon grids and stored in a vacuum desiccator for preservation before the measurement. TEM images for magnetite stock 2 were presented as Figure S2, while the quantification showed that ~90% of nanoparticles (474 grains in total) fell within the size range of 5 to 35 nm.

*Text S2. XANES measurement.* For $M_4$-edge HERFD measurement, U-containing magnetite wet pastes were separated by a strong magnet within the nitrogen-filled anoxic chamber, and then mounted into depressions (1-3 mm thick) within poly(methylmethacrylate) (PMMA) plates that were sealed independently with two layers of 8 μm-thick Kapton film. Sealed plates were immediately frozen inside a liquid nitrogen-cooled cold well inside the anoxic chamber and then stored in the freezer (-20 °C) inside the anoxic chamber until

transport to the MARS beamline in SOLEIL, France. The synchrotron was operated in top-up mode with an electron current of 500 mA. The incident beam at the MARS beamline is produced by a bending magnet source and selected by a double crystal monochromator (Si(111)). HERFD-XANES measurements were performed at the uranium $M_4$-edge. Potassium in KBr was used for energy calibration of the incident beam. The emission spectrometer was equipped with a bent Si(220) crystal analyzer (R=1m), and a KETEK single element silicon drift diode. The HERFD-XANES spectra were collected by setting the emission spectrometer at the maximum intensity of the uranium $M_\beta$ emission line. Sample plates were placed inside a cooling frame where the cryo-system (cryo-fluid at -25℃) was applied to keep samples frozen (~-18°C) during the measurement. The cooling stage, spectrometer crystal and detector were all placed inside a chamber filled with a He atmosphere for measurements.

The sample preparation for $L_3$-edge XANES measurements was conducted under anoxic conditions (3%: 97% $H_2$: $N_2$). The U-containing magnetite suspension was filtered through polyethersulfone membrane filters (0.22 μm) in Steriflip devices (Merck Millipore, Germany) or in a filtration unit to obtain solids on the filter. The filter holding solids was taped and enclosed with Kapton tape, and placed in Nalgene cryovials for measurement. Samples were shipped to Diamond Light Source (DLS) in a hermetically sealed stainless-steel shipping canister (Schuett-Biotec GmbH, Gottingen, Germany). The XAS spectra were collected in fluorescence mode at beamline (BL) B18. A 9-element Germanium (Ge) detector (Canberra industries, USA) was used to collect fluorescence signal. XAS analysis was conducted at 77 K in liquid nitrogen (LN2) cryostats to reduce beam damage, preserve air-sensitive samples from oxidation, and improve data quality. Energy calibration was performed on the first inflection point of the same yttrium (Y) foil reference (17,038 eV) for all samples during measurements at BL B18.

*Text S3. Isotope mass balance*. The mass balance was calculated for experiments where the isotopic signatures of both U-mag-bic-aq and *U-mag-bic-solid* were acquired (Figure S8). Among the seven batches, most data points achieved good isotopic mass balance, with the overall $\delta^{238}$U close to 0. However, positive $\delta^{238}$U values were observed for those experiments for which the sampling was performed by mixing a small volume (500 µL) with bicarbonate solution followed by direct filtration (6.25-B, 12.5-B, 25-A, 25-B). Deviation from mass balance is particularly pronounced when $C/C_0$ approached 0 and for the latter two experiments. We attribute this deviation to sampling artefacts. Sampling consisted of collecting a volume of suspension, amending with sodium bicarbonate and filtering. The filter was then washed with acid to recover solid-associated U. However, inevitably, some volume of liquid was retained in the filter, and as a result, is measured as part of the solid phase. For the 500-µL sample, the filter-retained volume represented a larger fraction of the total U than the ≥ 2-mL sample (collected for Exp. 25-PIPES and 25-noPIPES), resulting in accounting for U(VI) and U(V) twice: once in the filtrate (U-mag-bic-aq) and once in the retentate (*U-mag-bic-solid)*. A second difference in sampling between 25-PIPES/25-noPIPES and others (e.g. 25-A/25-B) was the use of a strong magnet to separate solid from liquid prior to filtration for the PIPES and no PIPES systems (Figure S1), resulting in better solid/liquid separation. Finally, for the 62.5-E, because the full reactor content was sacrificed at each time point to collect bicarbonate-extracted solution and the remaining solids, the solid sample volume was large, precluding its impact by residual solution and allowing for isotope mass-balance to be achieved.

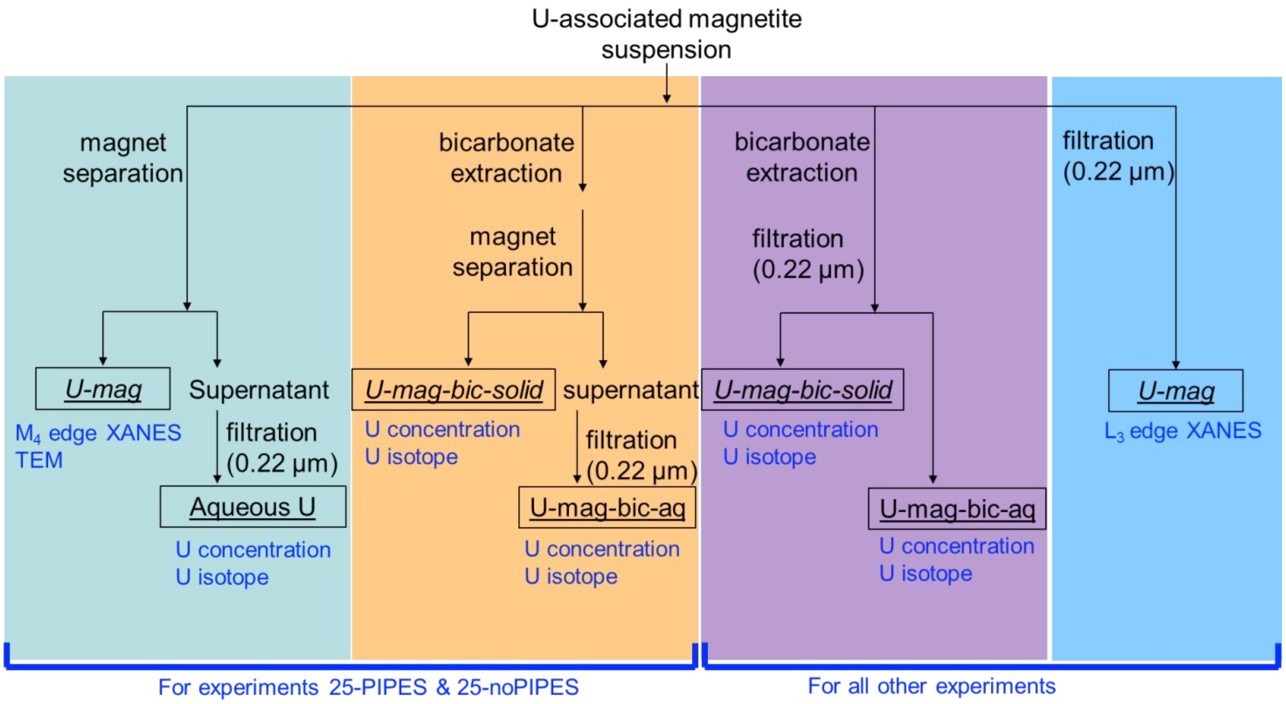


Figure S1. Schematic of experimental manipulations.

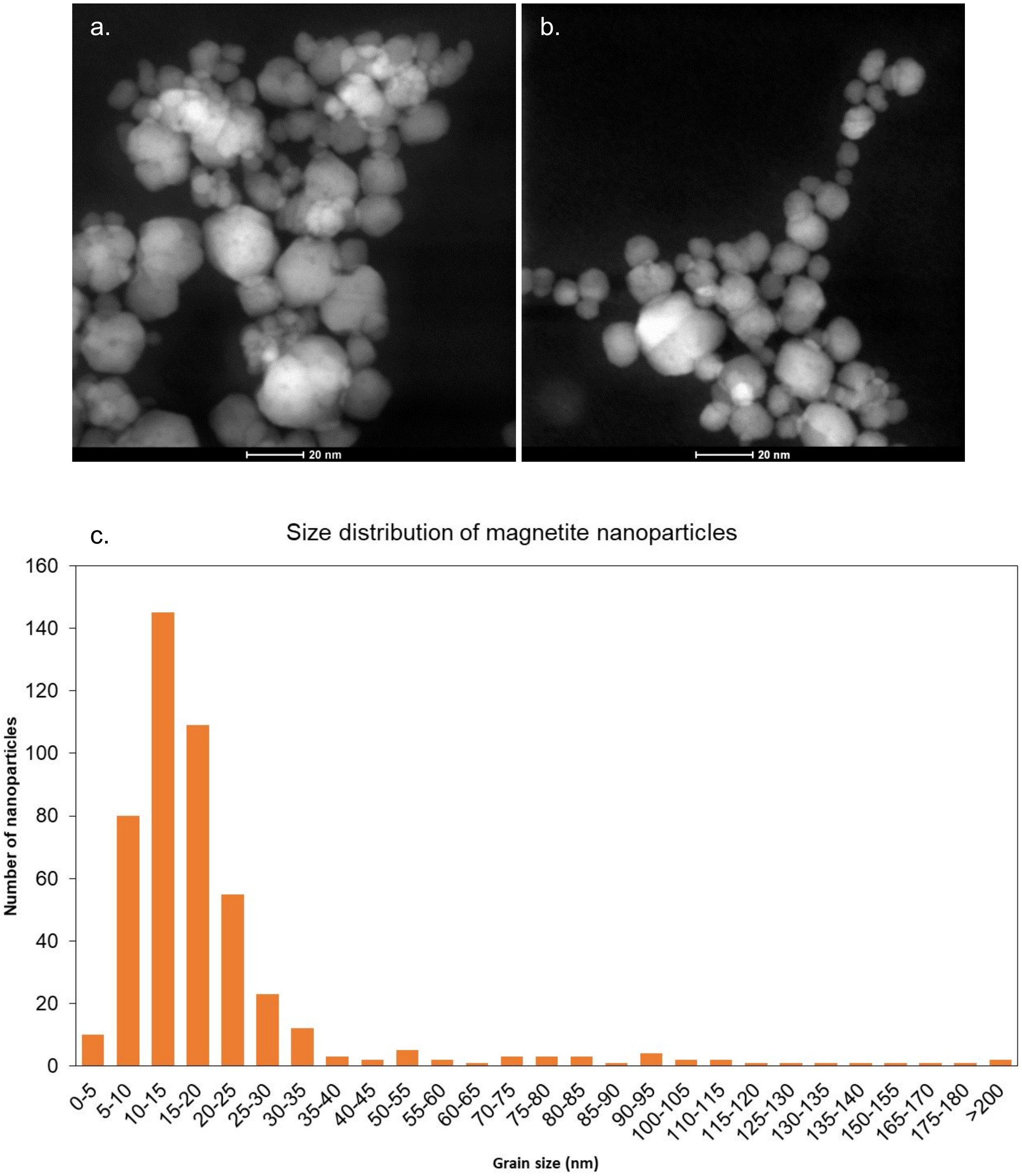


Figure S2. (a) and (b): TEM images of magnetite nanoparticles before reaction (Stock 2, lacey carbon grid). Scale bar: 20 nm; (c) size distribution of magnetite nanoparticles from Stock 2, 474 grains in total.

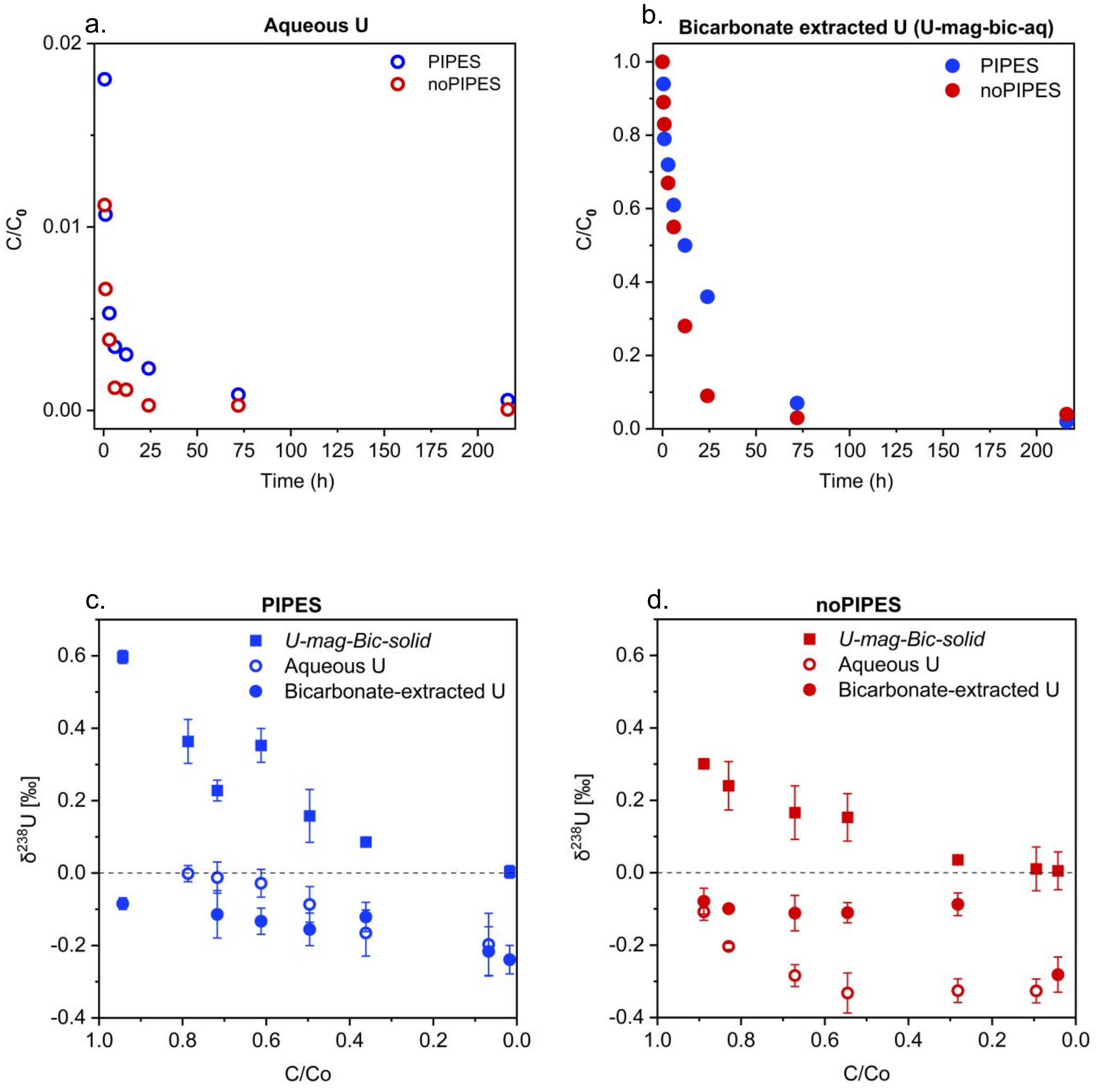


Figure S3. U(VI) reduction by magnetite in the presence and absence of 20 mM PIPES (Exp. 25-PIPES and 25-noPIPES). The concentration ($C/C_0$) of time-dependent aqueous U (open circles) (a) and bicarbonate-extracted U (U-mag-bic-aq, filled circles) (b) for U(VI) incubated with magnetite in 25-PIPES and 25-noPIPES experiments. A small contribution of aqueous phase U was also included in the bicarbonate extracted phase The isotope signatures ($\delta^{238}U$, ‰) of aqueous U (open circles), bicarbonate extracted U (U-mag-bic-aq, filled circles), and the remaining U in solid (*U-mag-bic-solid*, filled squares) for the system with PIPES (c) or without PIPES (no PIPES) (d). $C/C_0$ represents the ratio of the concentration of U that could be extracted by bicarbonate solution to the initial uranium concentration. Error bars in (c) and (d) represent isotopic measurement 2 S.D. values. The comparison of $\delta^{238}U$ values of aqueous U, U-mag-bic-aq or *U-mag-bic-solid* between PIPES and noPIPES systems are presented in Figure 2. The concentration (C) in $C/C_0$ always refers to bicarbonate-extractable U.

**A**

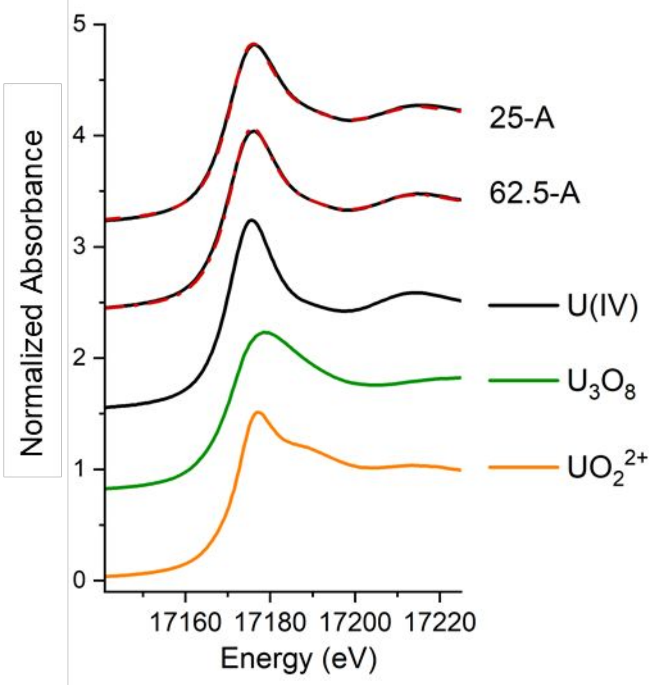


**B**

| | U concentration | U(IV) | $U_3O_8$ | U(VI) | R-factor | chi-square | reduced chi-square |
|---|---|---|---|---|---|---|---|
| | | % | % | % | | | |
| 25-A | 200 uM | 69.4(0.6) | 30.6(0.6)[a] 20.4 U(V) and 10.2 U(VI) | -[c] | 0.000718 | 0.02103 | 0.000155 |
| 62.5-A | 80 uM | 80.4(0.9) | 19.6(0.8)[b] 13.1 U(V) and 6.5 U(VI) | -[c] | 0.001130 | 0.0321 | 0.000236 |

a. 30.6% $U_3O_8$ can be converted to 20.4% U(V) and 10.2% U(VI).

b. 19.6% $U_3O_8$ can be converted to 13.1% U(V) and 6.5% U(VI).

c. '-' represents that standard (specifically adsorbed U(VI) on ferrihydrite in this case) is not required for the optimal linear combination fitting.

Figure S4. Bulk U $L_3$ edge XANES spectra and the results of the linear combination fit of magnetite-associated U in solid phase samples. (**A**) Normalized bulk U $L_3$ edge XANES spectra of *U-mag* samples (30 h) collected from Exp. 25-A and 62.5-A (black line) and the linear combination fitting (red dashed line), using U(VI) adsorbed on ferrihydrite as U(VI) standard (orange line), biogenic $UO_2$ as U(IV) standard (black line), and $U_3O_8$ as U(V)/U(VI) standard (green line). (**B**) Fit results are reported in the above Table S1 Values in parenthesis are the uncertainties of the LCF from Athena[3]. $U_3O_8$ reference compound consist of 2 U(V) and 1 U(VI)[4].

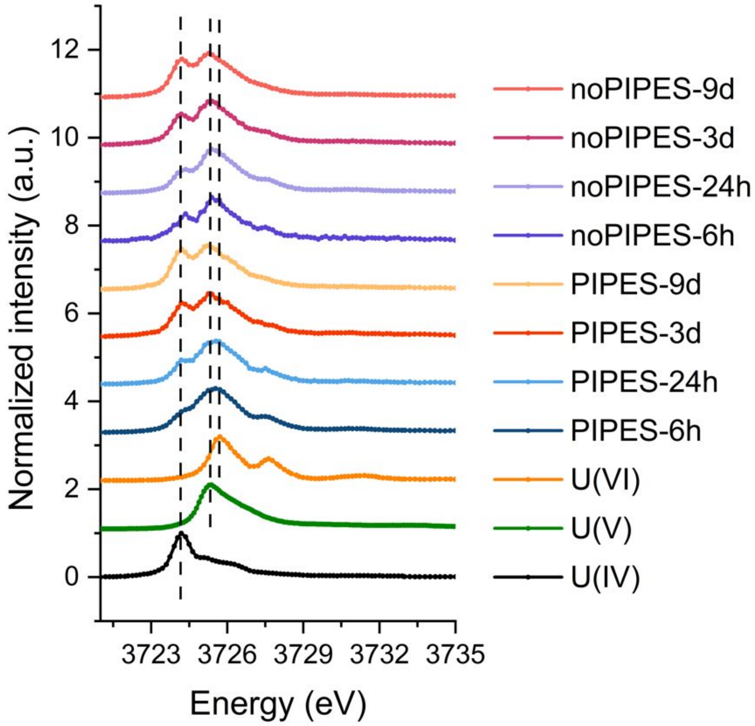


Figure S5. $M_4$-edge high-energy-resolution fluorescence detection X-ray absorption near-edge structure spectroscopy ($M_4$ edge HERFD-XANES) spectra for U-containing magnetite collected from 25-PIPES and 25-noPIPES systems after 6 h, 24 h, 3 d, and 9 d, compared to U(IV) ($UO_2$), U(V) (uranate in $UMoO_5$[2]), and U(VI) (uranyl(VI)-acetate) reference spectra. Dashed lines indicate the white line energy positions for U(IV), U(V), and U(VI) valence states, respectively. The spectra of PIPES and no-PIPES samples were interpreted with ITFA analysis and the results were presented in Figure 3.

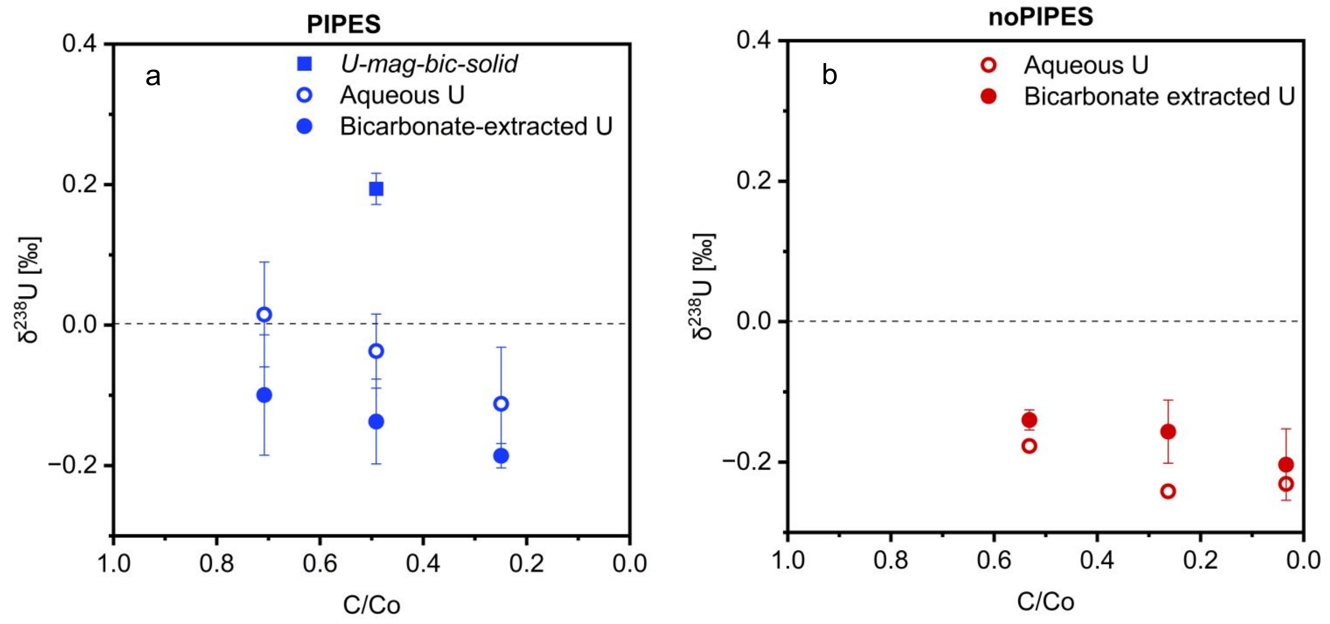


Figure S6. The isotope signatures ($\delta^{238}U$, ‰) of aqueous U (open circles), bicarbonate extracted U (U-mag-bic-aq, filled circles), and the remaining U in solid (*U-mag-bic-solid*, filled squares) of replicate experiments for system (a) with PIPES and (b) without PIPES (no PIPES) as Exp. 25-PIPES and 25-noPIPES. Aqueous phase or bicarbonate extracted phase at only three time points, and remaining solids after bicarbonate extraction at one time point were collected for isotope signature analysis, confirming the negative $\delta^{238}U$ values in aqueous phase and bicarbonate extracted phase, while the positive $\delta^{238}U$ value was again observed in the *U-mag-bic-solid* phase. Error bars represent isotopic measurement 2 S.D. values. The concentration (C) in $C/C_0$ always refers to bicarbonate-extractable U. Similar data (from a replicate experiment) are displayed in Figure 2.

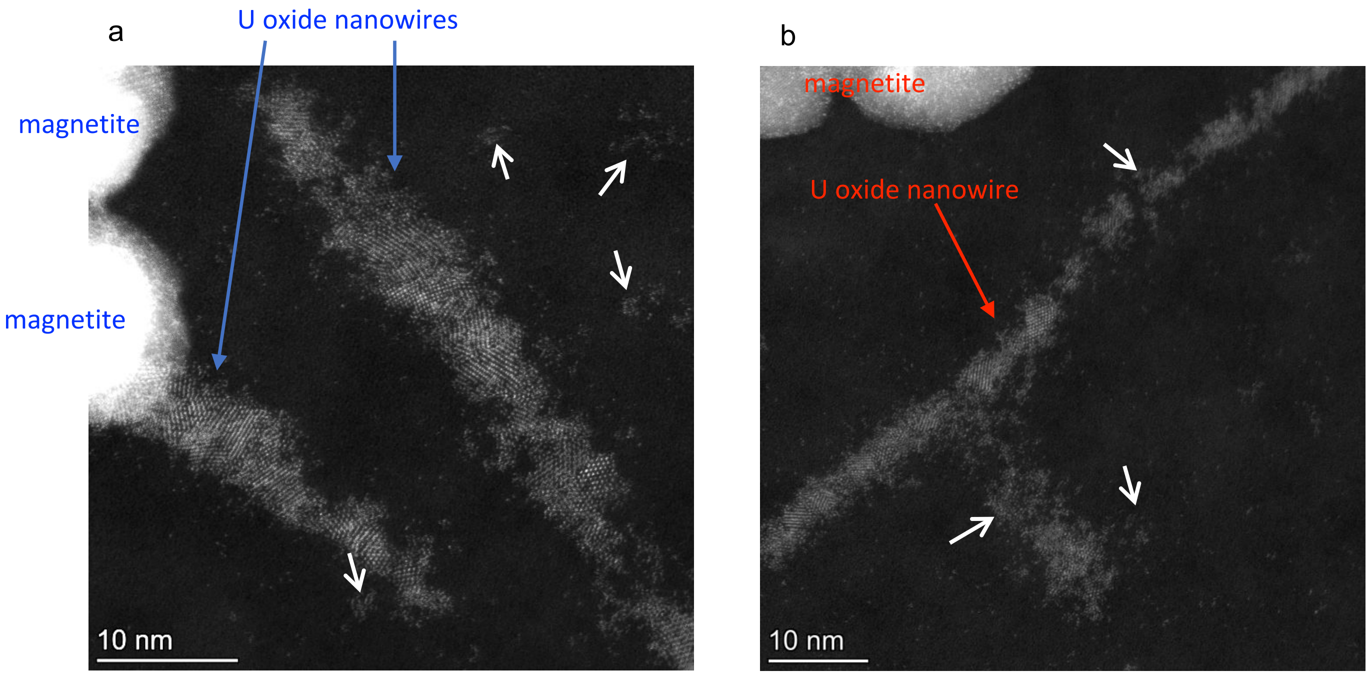


Figure S7. Two high-angle annular dark-field scanning transmission electron microscopy images of U-magnetite samples were obtained for Exp. 25-PIPES (a) and Exp. 25-noPIPES (b) at 24 h. Arrows point to dispersed amorphous clusters dispersed.

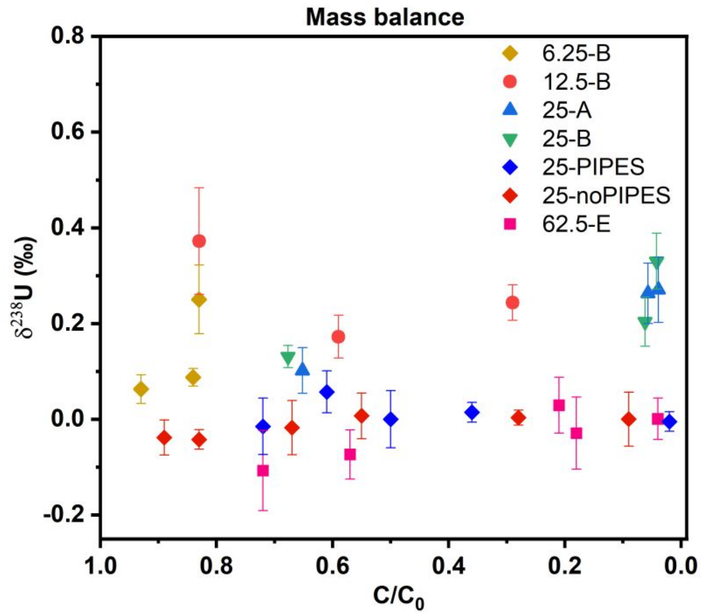


Figure S8. The overall isotope signatures ($\delta^{238}U$, ‰) acquired from isotopic mass balance calculations, each data point was calculated from $\delta^{238}U$ values for U-mag-bic-aq (Fig. 2) and *U-mag-bic-solid* (the corresponding solid phase after bicarbonate extraction in Fig. 3). Error bars represent the range of $\delta^{238}U$ values at each time points. The concentration (C) in $C/C_0$ always refers to bicarbonate-extractable U.

References


(1) Wang, Y.; Morin, G.; Ona-Nguema, G.; Menguy, N.; Juillot, F.; Aubry, E.; Guyot, F.; Calas, G.; Brown Jr, G. E. Arsenite Sorption at the Magnetite–Water Interface during Aqueous Precipitation of Magnetite: EXAFS Evidence for a New Arsenite Surface Complex. *Geochim. Cosmochim. Acta* **2008**, *72* (11), 2573–2586.

(2) Pan, Z.; Bártová, B.; LaGrange, T.; Butorin, S. M.; Hyatt, N. C.; Stennett, M. C.; Kvashnina, K. O.; Bernier-Latmani, R. Nanoscale Mechanism of UO2 Formation through Uranium Reduction by Magnetite. *Nat. Commun.* **2020**, *11* (1), 1–12. https://doi.org/10.1038/s41467-020-17795-0.

(3) Ravel, B.; Newville, M. ATHENA, ARTEMIS, HEPHAESTUS: Data Analysis for X-Ray Absorption Spectroscopy Using IFEFFIT. *J. Synchrotron Radiat.* **2005**, *12* (4), 537–541. https://doi.org/10.1107/S0909049505012719.

(4) Leinders, G.; Bes, R.; Pakarinen, J.; Kvashnina, K.; Verwerft, M. Evolution of the Uranium Chemical State in Mixed-Valence Oxides. *Inorg. Chem.* **2017**, *56* (12), 6784–6787. https://doi.org/10.1021/acs.inorgchem.7b01001.
